# Application of cholecystic duct plasty in the prevention of biliary complications following orthotopic liver transplantation

**DOI:** 10.3389/fsurg.2023.1087327

**Published:** 2023-05-03

**Authors:** Jing Wang, Song-ping Cui, Shao-cheng Lyu, Qing Chen, Jin-can Huang, Han-xuan Wang, Qiang He, Ren Lang

**Affiliations:** ^1^ Department of Hepatobiliary and Pancreaticosplenic Surgery, Beijing ChaoYang Hospital, Capital Medical University, Beijing, China; ^2^ Department of Thoracic Surgery, Beijing ChaoYang Hospital, Capital Medical University, Beijing, China

**Keywords:** orthotopic liver transplantation, biliary reconstruction, cholecystic duct plasty, biliary complication, prognosis

## Abstract

**Background:**

The purpose was aimed to evaluate the safety and effectiveness of cholecystic duct plasty (CDP) and biliary reconstruction techniques preventing biliary complications following orthotopic liver transplantation (OLT) first proposed by our center.

**Methods:**

127 enrolled patients who underwent LT in our center from January 2015 to December 2019 were analyzed retrospectively. According to the mode of biliary tract reconstruction, patients were divided into CDP group (Group 1, *n* = 53) and control group (Group 2, *n* = 74). The differences of perioperative general data, biliary complications and long-term prognosis between two groups were compared and analyzed.

**Results:**

All patients completed the operation successfully, the incidence of perioperative complications was 22.8%. There was no significant difference in perioperative general data and complications between the two groups. Follow-up ended in June 2020, with a median follow-up period of 31 months. During the follow-up period, biliary complications occurred in 26 patients, with an overall incidence of 20.5%. The overall incidence of biliary complications and anastomotic stenosis in Group 1 was lower than that in Group 2 (*P *< 0.05). There was no significant difference in overall prognosis between the two groups (*P* = 0.274), however, the cumulative incidence of biliary complications in Group 1 was lower than that in Group 2 (*P* = 0.035).

**Conclusion:**

Reconstruction of common bile duct by CDP represents considerable safety and practicability, particularly for patients with small diameter of common bile duct or wide discrepancy of bile duct size between donor and recipient.

## Introduction

After nearly 60 years of development, orthotopic liver transplantation (OLT) has become the best treatment for patients with end-stage liver disease and primary liver carcinoma that meet certain criteria, especially with the advent of immunosuppressive drugs such as cyclosporine and tacrolimus, the overall 5-year survival rate after OLT represents more than 70% (1, 2). With the prolongation of patients' survival, biliary complications have become one of the important risk factors affecting patients' postoperative quality of life, known as the “Achilles heel” of OLT, with an incidence of about 5%–32% and a mortality rate of about 10% (3, 4).

Despite the ongoing advancements in surgical techniques leading to a reduction in the incidence of biliary complications post OLT over the years, they remain the primary cause of complications and mortality following OLT (5, 6). Common biliary complications after OLT mainly include biliary leakage, anastomotic stricture, non-anastomotic stricture, bile duct stones, bile tumor, biliary bleeding, biliary casting syndrome and so on, meanwhile, the main risk factors include bile duct diameter, anastomosis technique, ischemia-reperfusion injury, ABO blood group incompatibility, hepatic artery complications and cytomegalovirus infection, among them, factors associated with surgical procedures include inappropriate surgical techniques (such as excessive dissection of peri-biliary tissue and excessive use of electrosurgery), inappropriate suture materials, mismatch between donor and recipient bile duct diameter, small diameter of donor bile duct (<4 mm), and excessive anastomotic tension (7, 8). biliary complications not only affect the living quality of OLT recipients, but leading to surgical failure and re-transplantation as well. Hence, the safe implementation of biliary reconstruction and prevention of biliary complications remain the primary focus of clinical research for patients with narrow biliary duct diameter and donor-recipient bile duct diameter mismatch.

The application of cholecystic duct plasty (CDP) in liver transplantation is a relatively new research field. CDP expands the bile duct diameter by trimming the connection between the cystic duct and the common bile duct, facilitating bile duct anastomosis and promoting bile drainage. This reduces the incidence of bile stasis and other complications, and improves the success rate of liver transplantation. In our center, we employed CDP when the common bile duct of the donor or recipient is too narrow, or when there is a diameter discrepancy of more than 1/2 between the donor and recipient bile ducts.

This research retrospectively analyzed the clinical data of 53 OLT patients who underwent CDP for biliary reconstruction in our center, in order to explore the safety and effectiveness of this novel technique for preventing biliary complications following OLT.

## Materials and methods

### Ethics approval and consent to participate

The study was conducted in accordance with the Declaration of Helsinki (as revised in 2013) and was approved by the Ethics Committee of Beijing Chaoyang Hospital (No. 2020-D.-304). All recipients in this study received livers from donors post circulatory death (DCD). Participant informed consent was exempted because of the retrospective study design, and the study design was approved by the appropriate ethics review board.

### Patients' selection and preoperative characteristics

Data of patients undergoing OLT admitted to our hospital from January 2015 to December 2019 were retrospectively analyzed. According to relevant inclusion/exclusion criteria, a total of 127 eligible OLT patients were selected for analysis. The authors take full responsibility for all aspects of the work, ensuring that any questions regarding the accuracy or integrity of any part of the work are thoroughly investigated and resolved.

### Inclusion and exclusion criteria

Inclusion criteria: (1) patients underwent OLT from January 2015 to December 2019. (2) No restriction was imposed on age and gender. (3) All patients were treated with orthotopic liver transplantation. (4) Clinical and follow-up data of the patients were complete. The diameter of the common bile duct was measured directly by the surgeon during surgery. If the diameter of the donor or recipient's common bile duct is less than 6 mm, or if the diameter of the donor and recipient's common bile duct differs by more than 1/2, CDP will be performed.

Exclusion criteria: (1) perioperative death. (2) Patients with cholecystectomy. (3) Previous history of biliary tract-related surgery (biliary exploration, ERCP, ENBD, etc). (4) Patients with choledocholithiasis, cholecystolithiasis and other biliary diseases. (5) Patients with autoimmune liver diseases such as autoimmune hepatitis (AIH), primary biliary cirrhosis (PBC) and primary sclerosing cholangitis (PSC).

In the present study, all recipients received livers from DCD donors. The selection criteria for the donor liver included: (1) age, generally no limitation. (2) Clinical history, previous viral, alcoholic or fatty liver disease, history of hepatobiliary surgery, uncontrolled abdominal infection, history of alcoholism and liver trauma are usually considered as risk factors for poor prognosis of liver transplantation. (3) Liver function, including transaminases, bilirubin, alkaline phosphatase, lactate dehydrogenase (LDH), albumin and coagulation tests, non-liver-derived factors should be excluded based on clinical history. (4) Liver morphology, by liver ultrasonography to rule out significant fatty liver, cirrhosis, fibrosis or other morphological abnormalities. (5) Macroscopic observation and perfusion of the liver, observation of liver color and changes in the liver before and after perfusion. Usually significant liver fibrosis, cirrhosis, or fatty liver is not transplantable (9).

A total of 127 patients, comprising 86 males and 41 females with a male-to-female ratio of 1.3:1, met our inclusion criteria. Their ages ranged from 21 to 73 years old, with a mean age of 50.1 ± 10.5 years. The main causes include 54 cases of liver failure (37 cases of hepatitis cirrhosis, 7 cases of alcoholic liver cirrhosis, 7 cases of drug-induced liver damage, 3 cases of autoimmune liver disease), 38 cases of primary liver malignant tumor [19 cases in accordance with Milan criteria, 29 cases in accordance with Hangzhou criteria (10)], 31 cases of liver cirrhosis (24 cases caused by hepatitis virus, 5 cases by alcohol and 2 cases by autoimmunity). Liver re-transplantation caused by chronic liver rejection in 3 cases and polycystic liver in 1 case. Of all the patients, 82 (64.6%) cases had a history of hepatitis B, 16 (12.6%) cases had a history of hepatitis C, and 72 (56.7%) cases had a previous history of esophagogastric variceal hemorrhage. Among the 89 patients with benign disease, Child grading included: 3 cases of GRADE A, 35 cases of GRADE B, 51 cases of GRADE C and model of end-stage liver disease (MELD) score was 7–46 (18.9 ± 8.9).

### Patients grouping and definition

**Figure 1:**
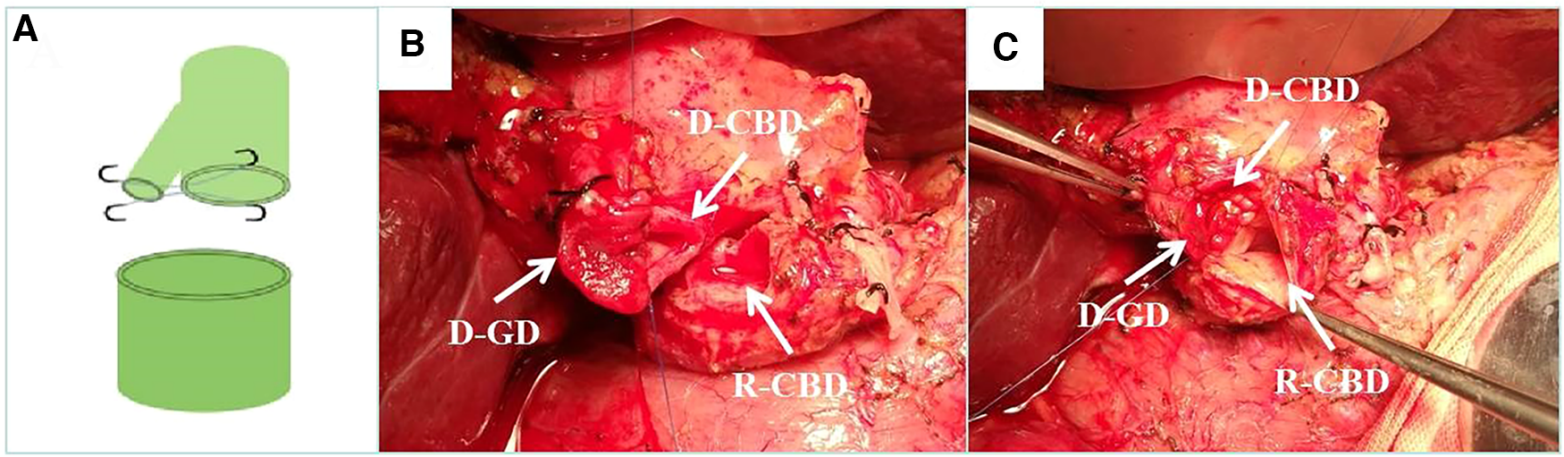
Donor cholangioplasty. (**A**) Diagram of donor cholangioplasty pattern. (**B**) Intraoperative donor cystic duct with choledochoplasty. (**C**) End-to-end anastomosis with the recipient common bile duct after intraoperative donor cholangioplasty. D-CBD, Donor Common Bile Duct; D-GD, Donor cystic duct; R-CBD, Recipient Common Bile Duct.

**Figure 2:**
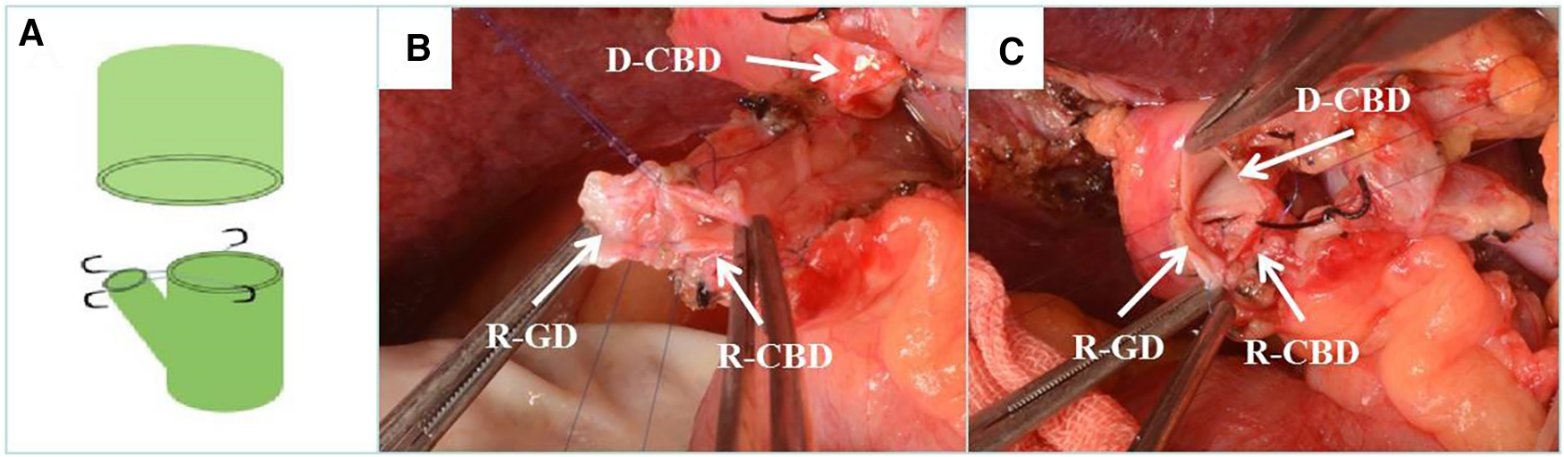
Recipient cholangioplasty. (**A**) Diagram of recipient cholangioplasty pattern. (**B**) Intraoperative recipient cystic duct and choledochoplasty. (**C**) End-to-end anastomosis with donor common bile duct after intraoperative recipient cholangioplasty. D-CBD, Donor Common Bile Duct; R-GD, recipient cystic duct; R-CBD, recipient Common Bile Duct.

**Figure 3:**
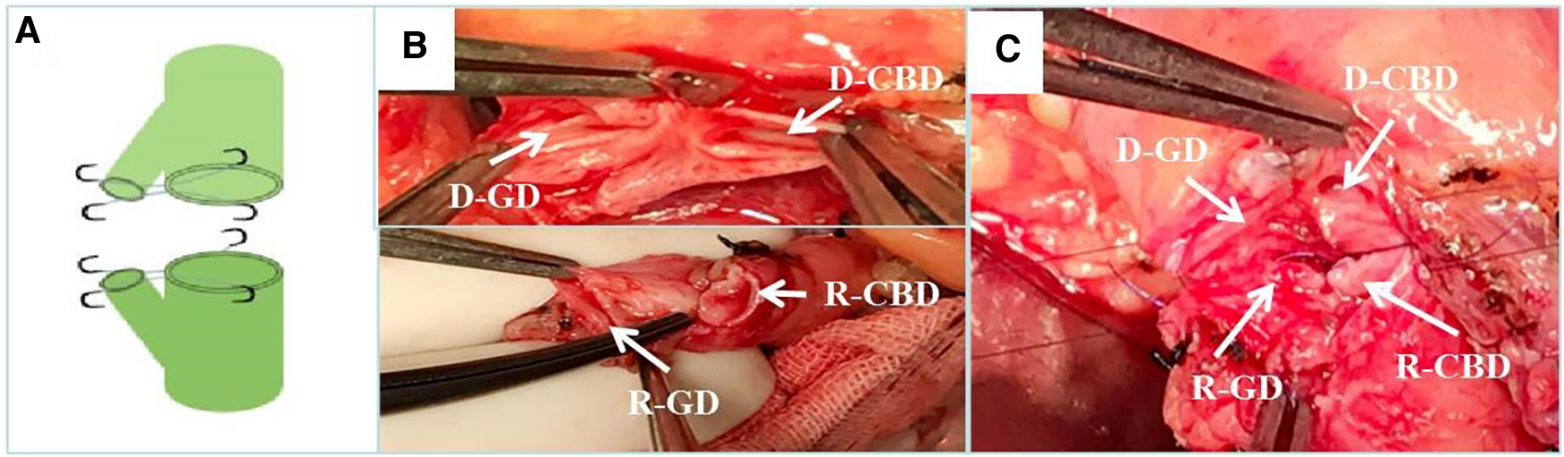
Donor-recipient cholangioplasty. (**A**) Diagram of donor and recipient cholangioplasty pattern. (**B**) The cystic duct and common bile duct of the donor and recipient are formed separately during surgery. (**C**) end-to-end anastomosis after intraoperative donor and recipient cholangioplasty. D-CBD, Donor Common Bile Duct; D-GD, Donor cystic duct; R-CBD, recipient Common Bile Duct; R-GD, recipient cystic duct.

According to the mode of biliary tract reconstruction, patients were divided into CDP group (Group 1, *n* = 53) and control group (Group 2, *n* = 74). In Group 2, the common bile duct was anastomosed end-to-end with 6-0 absorbable suture, continuous posterior wall and intermittent anterior wall for biliary duct reconstruction. In the Group 1, according to the diameter and ratio of the common bile duct between the donor and recipient, for the thinner side, the cystic duct was reserved in advance during dissociation. During biliary reconstruction, the common bile duct and the cystic duct were cut open to be close to the medial wall. The 7-0 prolene line was used to suture the medial wall of the common bile duct and cystic duct intermittently. Forming an opening between the cystic duct and the common bile duct, and then make an end-to-end anastomosis with the other side of the common bile duct. To sum up, Group 1 were able to be subdivided into three types according to the specific shaping methods: donor choledochoplasty (Figure 1), recipient choledochoplasty (Figure 2), donor-recipient choledochoplasty (Figure 3). T tube was not placed in this group.

### Surgical details and perioperative treatment

When removing the diseased liver, the cystic duct was divided 1–2 cm away from the common bile duct, and the common hepatic duct was severed above the confluence level of the cystic duct. And the specific CDP and biliary reconstruction techniques were summarized as follows:

(1) Choledochoplasty of cystic duct—common bile duct on the donor side:
(2) Choledochoplasty of gallbladder neck—common bile duct on the donor side:
(3) Choledochoplasty of gallbladder neck-cystic duct—common bile duct on the donor side:
(4) Choledochoplasty of cystic duct—common hepatic duct on the recipient side:

The standard triple regimen of prednisone + tacrolimus/cyclosporine + mycophenolate mofetil was employed as postoperative immunosuppressant therapy. The specific drug regimen was modified based on monitoring of postoperative liver function and drug concentration, in order to maintain continuous improvement and stability of liver function within the normal range.

### Follow up strategy and index analysis

Our follow up was finished in June 2020 and the medium follow-up period was 31 months. The perioperative information and postoperative recovery condition of enrolled patients (including gender, age, history of hepatitis, etiology of transplantation, MELD score, Child grade, operation time, cold ischemia time, blood loss, blood transfusion or not, immunosuppression program and biliary complications) were obtained from medical records and were compared between different groups. All patients undergo regular physical examination, drainage fluid and peripheral blood tests, and abdominal CT in the early postoperative period. If any abnormalities are detected, relevant tests are conducted promptly to assess the presence of any associated complications. The diagnosis of biliary complications was made by two experienced hepatobiliary surgeons based on previous literature. We scheduled follow-up for the 1st and the 3rd month within the first 3 months. Then we arranged follow-up every 3 months within the first 2 years after operation and every 6 months after 2 years. Tumor recurrence and death of patients indicated the endpoints of follow-up visits.

### Statistical analysis

Measurement data fitting normal distribution were expressed as mean ± standard deviation while data fitting non-normal distribution were expressed as median (interquartile range). *T*-test was adopted for normal distribution and rank sum test was used for non-normal distribution when comparing measurement data between the two groups. Chi-square test was used to compare the counting data between two groups and Fisher's exact probability method was used when the theoretical frequency was less than 1. The survival curve was calculated using the Kaplan-Meier method and evaluated with the log-rank test. Statistical significance was defined as *P* < 0.05 and all data were analyzed by SPSS for Macintosh (version 24.0; IBM, Armonk, NY).

## Results

### Surgical outcomes

All patients completed the operation successfully, the intraoperative blood loss was 600 (500, 800) ml, blood transfusion was performed in 65 cases (51.2%), and the operation time was 6.2–13.3 (9.2 ± 1.7) hours. Out of the 53 patients who underwent CDP, donor choledochoplasty was carried out in 36 cases, recipient choledochoplasty in 11 cases, and donor-recipient choledochoplasty in 6 cases.

### Comparison of perioperative data between two groups

The comparison of perioperative baseline data between two groups was shown in Table 1, which indicated that there was no statistically significant difference in baseline data between two groups (*P* > 0.05). Postoperative hospital stay ranged from 13 to 42 (22.3 ± 5.2) days, meanwhile, perioperative complications occurred in 29 patients, with a morbidity rate of 22.8%, the details of perioperative complications were shown in Table 2 and there was no significant difference in between two groups (*P* > 0.05).

**Table 1:** Comparison of perioperative baseline data between patients in plastic control group.

Variable	CDP group (*n* = 53)	Control group (*n* = 74)	*P* value
Gender (M/F)	37/16	49/25	0.669
Age (year)	49.6 ± 11.1	50.4 ± 10.1	0.677
Pathology (benign/malignant)	38/15	51/23	0.736
Types of liver disease			0.157
Liver failure	20	34	
Primary liver malignant tumor	15	23	
Liver cirrhosis	16	15	
Others	2	2	
History of hepatitis (yes/no)	38/15	60/14	0.214
Esophagogastric variceal hemorrhage (yes/no)	33/20	39/35	0.284
Child classification (A/B/C)	12/22/19	22/20/32	0.228
MELD score	18.0 ± 7.7	16.3 ± 10.0	0.288
Milan criteria (yes/no)	7/8	12/11	0.740
Hangzhou criteria (yes/no)	12/3	17/6	0.666
Donor common bile duct diameter (mm)	5.4 ± 1.4	5.8 ± 1.5	0.130
Recipient common bile duct diameter (mm)	7.2 ± 1.3	7.7 ± 2.3	0.156
Intraoperative bleeding (ml)	600 (500, 800)	600 (500, 800)	0.112
Intraoperative transfusion (yes/no)	26/27	39/35	0.685
Procedure duration (h)	9.3 ± 1.5	9.2 ± 1.7	0.775
Cold ischemia time (h)	8.6 ± 2.1	8.3 ± 2.7	0.698
Warm ischemia time (min)	46.8 ± 10.9	50.2 ± 12.5	0.114
Immunosuppressive regimen (tacrolimus/cyclosporine)	42/11	62/12	0.513

**Table 2:** Comparison of perioperative complications between patients in the plastic control group.

Complication	CDP group (*n* = 53)	Control group (*n* = 74)	*P* value
Acute rejection	3	5	0.905
Acute kidney injury	2	3	0.702
Lung infection	2	4	0.997
Abdominal infection	1	2	0.769
Hemoperitoneum	2	3	0.702
Biliary fistula	1	4	0.587
Graft-vs.-host disease	1	1	1.000
Hepatic artery thrombosis	0	1	1.000

### Comparison of long-term prognostic data in different groups

**Figure 4:**
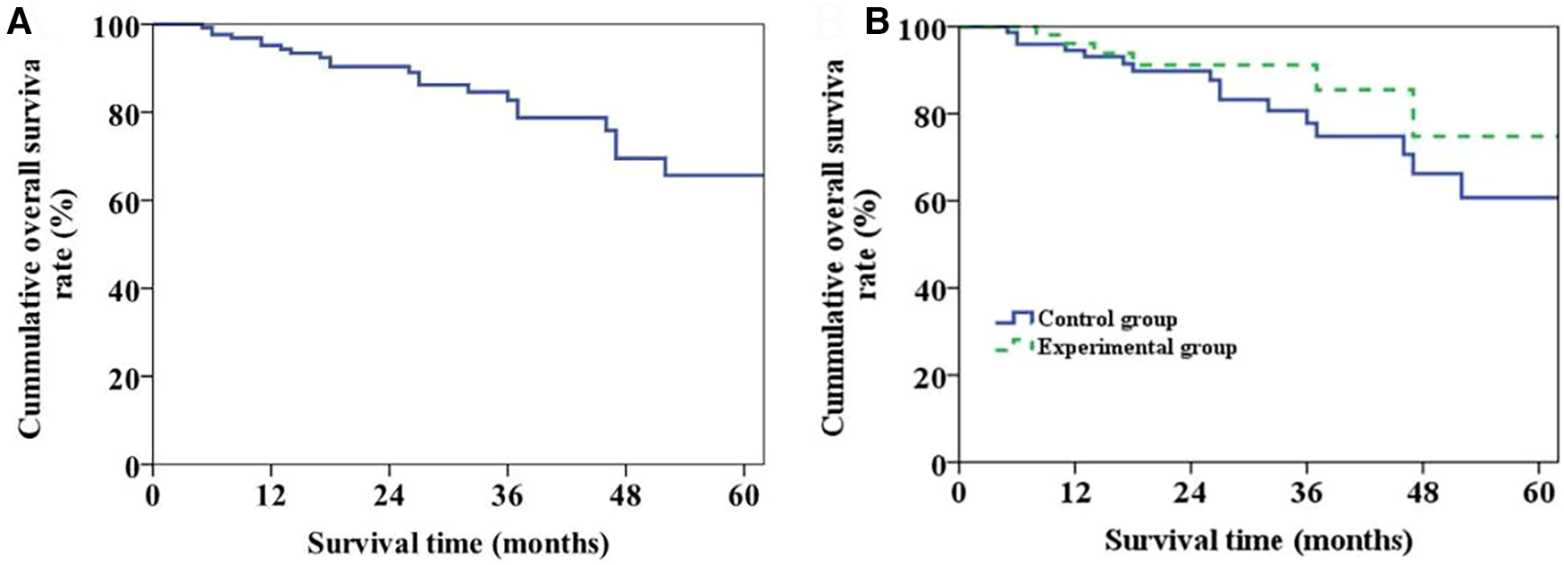
Long-term survival of patients. (**A**) Plot of overall long-term survival curve of patients. (**B**) Long-term survival curve of patients in the two groups (*P* = 0.274).

Throughout the follow-up period, a total of 22 patients passed away, with 10 cases attributed to tumor recurrence, 8 cases linked to postoperative infection (including 5 cases of pulmonary infection and 3 cases of biliary tract infection), 3 cases due to chronic rejection, and 1 case caused by cerebral hemorrhage. The overall survival curve of the patients was shown in Figure 4A. The 1-, 3- and 5-year survival rates were 95.2%, 82.7% and 65.7%, respectively. The overall survival curves of two groups were shown in Figure 4B. The 1-, 3- and 5-year survival rates of patients in Group 1 and 2 were 96.1%, 85.5%, 74.8% and 94.5%, 77.8%, 60.7%, respectively (*P* = 0.274).

### Differences of biliary complications between two groups

**Figure 5:**
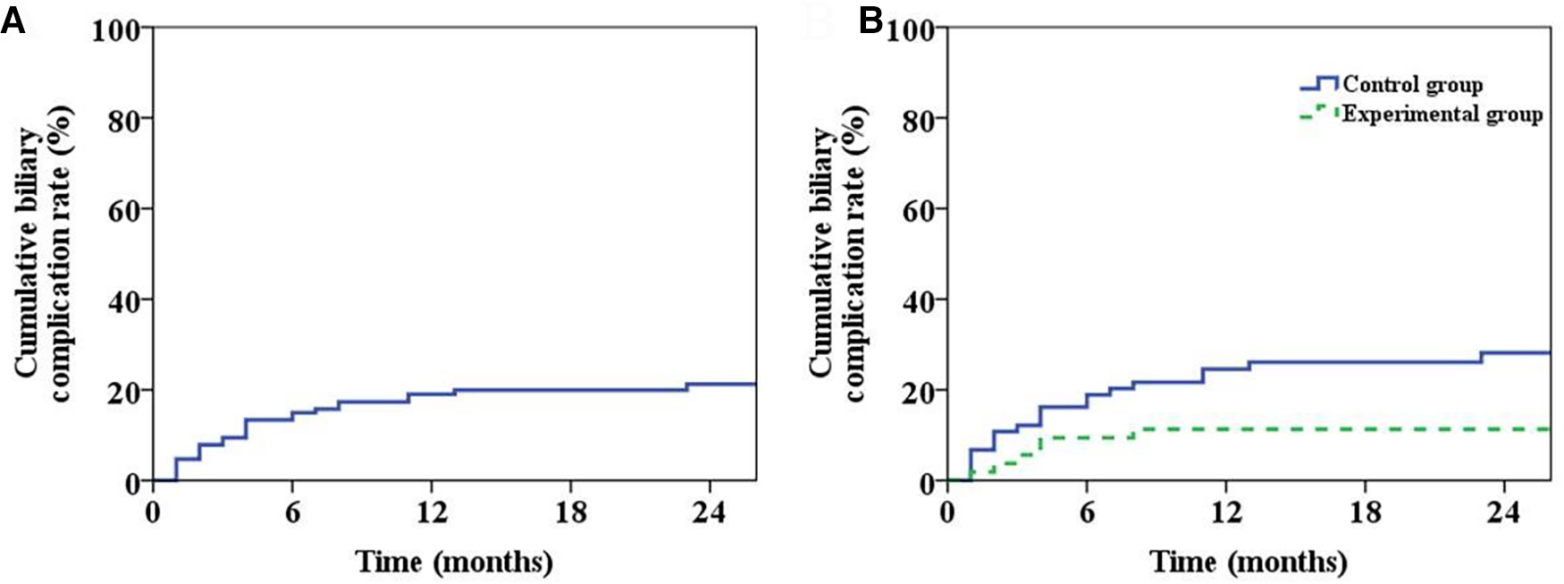
Patient biliary complications. (**A**) The graph of overall incidence of biliary complications in patients. (**B**) The incidence curve of biliary complications in the two groups (*P* = 0.035).

During the follow-up period, 26 patients developed biliary complications, with an overall incidence of 20.5%. Meanwhile, the incidence of postoperative biliary complications and anastomotic stenosis in Group 1 was lower than that in Group 2 (*P* < 0.05) (Table 3). The overall biliary complication curve of the patient was shown in Figure 5A and the incidence rates of half a year, 1 year and 2 years after operation were 15.0%, 19.0% and 21.2%, respectively. The occurrence curve of biliary complications in two groups was shown in Figure 5B while the incidences of half a year, one year and two years after operation in Group 1 and Group 2 were 9.4%, 11.3%, 11.3% and 18.9%, 24.6%, 28.2%, respectively (*P* = 0.035).

**Table 3:** Comparison of biliary complications between patients in the plastic control group.

Variable	CDP group (*n* = 53)	Control group (*n* = 74)	*P* value
Anastomotic stenosis	3	13	0.046
Non-anastomotic stricture	1	1	1.000
Biloma	1	2	0.769
Biliary fistula	1	4	0.587
Bile duct stone	2	7	0.379
Hemobilia	0	1	1.000
Bile duct mucocele	0	1	1.000
Biliary complications	6	20	0.031

Out of the patients who experienced early postoperative biliary fistula, 5 were treated with biliary fistula repair. Additionally, 2 patients developed biliary anastomotic stricture in the long term after surgery. Furthermore, 1 patient suffered from early postoperative biliary bleeding and required laparotomy and biliary hemostasis. 3 patients with choleoma were found early after operation, among them, 2 patients underwent ERCP stent implantation and 1 patient received oral drug therapy only. Meanwhile, among the 16 patients with long-term postoperative biliary anastomotic stenosis, 9 patients were complicated with bile duct stones, 13 patients received ERCP balloon dilation & stent implantation, 2 patients received ERCP balloon dilation alone, and 1 patient received oral drug therapy only. 2 patients with long-term postoperative non-anastomotic stenosis received oral drug therapy. Of the 26 patients with biliary complications, 3 died of biliary infection and 1 underwent secondary liver transplantation due to liver decompensation.

## Discussion

Despite the rapid progress in liver transplantation-related techniques, the incidence of biliary complications has remained high in recent years, about 30% of the patients after transplantation have biliary complications, including bile leakage, anastomotic stricture, non-anastomotic stricture, bile duct stones, etc., which has become one of the important factors seriously affecting the survival rate and quality of life of OLT patients (2).

According to the different time period after OLT, the specific types of biliary complications are also different. Sixty percent of biliary complications following OLT occur within the first three months after the operation, with ninety percent occurring within one year. Two most common biliary complications are bile leakage and anastomotic stricture. The incidence of bile leakage after OLT is 20% and 27% of patients will have late anastomotic stricture. Although there are many factors affecting anastomotic stricture and bile leakage following OLT, such as cold ischemia time, chronic rejection, hepatic artery thrombosis and other donor and recipient factors, anastomotic stenosis and bile leakage caused by anastomotic technique are recognized as the main causes (11). Previous studies have shown that recipients of livers from DCD donors have an increased incidence of biliary complications, primary nonfunction (PNF), and hepatic artery thrombosis, and therefore poorer long-term outcomes compared with donors donation after brain death (DBD), with a 30% incidence of ischemic cholangiopathy (IC) for DCD LT and 2%–4% for DBD LT (12–16). There is a strong correlation between factors such as the presence of warm ischemia-induced damage before cold ischemia during DCD (Donation after Circulatory Death) donor acquisition, blood stasis during warm ischemia, and microthrombosis of the microcirculation around the bile duct (17–20). The national results of DCD LT are improving with the in-depth study of the mechanisms of DCD LT-related injuries and the practice of prevention strategies including improvement of procurement based (super rapid technique, minimizing warm ischemia time, and so on), minimizing cold ischemia time, machine perfusion, etc (21, 22). In this study, 26 patients developed biliary complications during the follow-up period, with an overall incidence of 20.5%. The incidence of postoperative biliary complications and anastomotic stenosis in CDP group was lower than that in control group (*P* < 0.05), 11.3% vs. 27.0%, 5.7% vs. 17.6%, respectively. This implies that reconstruction of the common bile duct by CDP may be effective in preventing biliary complications after OLT.

Kaldas et al. (23) conducted univariate analysis and logistic multivariate analysis on 503 patients after OLT showed that biliary reconstruction was one of the independent risk factors for biliary complications of anastomosis. For patients with thin diameter of common bile duct, if the suture is too dense, it is easy to cause anastomotic stricture and ischemia due to the traction and contraction effect of suture after knotting; if the suture is too sparse, it is easy to cause biliary fistula. For patients with excessive difference in the diameter ratio of common bile duct between donor and recipient, stenosis is more likely to occur on the thinner side after suture, and biliary fistula is more likely to occur on the wider side. Hence, the safe execution of biliary reconstruction and the prevention of associated complications in such patients have become a crucial area of focus in clinical research.

It has been reported that biliary stricture after liver transplantation can be prevented by placement of a T-tube, longitudinal incision and plasty of the biliary wall, and choledochojejunostomy, but this is still controversial (24). In the early stage of living donor liver transplantation, choledochojejunostomy is the standard for bile duct reconstruction and is still used. However, Roux-en-Y choledochojejunostomy has its inherent disadvantages, such as easy contamination of the abdominal cavity by jejunal contents during surgery, long operation time, and delayed recovery of intestinal function, and choledochojejunostomy abandons the normal biliary system anatomy, abandons the use of the sphincter of Oddi, and predisposes to biliary complications such as cholangitis and bile duct stones after surgery, which is not conducive to the postoperative application of endoscopic methods such as ERCP to treat biliary complications (25, 26). Therefore, our center does not routinely use choledochojejunostomy technique during surgery and only recommends it when a duct to duct anastomosis is not possible or appropriate.

Whether T-tube placement in liver transplantation can reduce postoperative biliary complications is not clear. Weiss et al. (27) showed through a randomized controlled study of 194 patients that T-tube placement in liver transplantation can significantly reduce the overall incidence of complications (27% vs. 53%, *P* < 0.05), but did not reduce the incidence of complications of biliary fistula and anastomotic stricture. Scatton et al. (28) through a multicenter randomized controlled study of 180 patients, showed that the incidence of biliary complications was significantly higher in patients with intraoperative T-tube placement than in those without T-tube placement (33.3% vs. 15.5%, *P* < 0.05). Sun et al. (29) performed a meta-analysis of six randomized controlled studies on whether a T-tube was placed during liver transplantation and found that T-tube placement during liver transplantation did not reduce the incidence of postoperative biliary complications. Moreover, the removal of the T-tube within 3–6 months following surgery is mandatory after its placement, which not only imposes additional challenges for patients but also carries a risk of biliary fistula development. Therefore, our center does not routinely place T-tube during surgery, but for patients who undergo reoperation due to postoperative biliary complications such as biliary fistula and anastomotic stricture, because most of the local biliary tract inflammation affects healing at this time, our center recommends the placement of T-tube to prevent stricture.

A longitudinal incision and plasty of the biliary wall has been reported in the literature to have a positive impact on preventing biliary complications (30). However, for patients with large difference in the diameter of donor and recipient common bile duct, if the longitudinal incision of common bile duct is too long, bile fistula and local biloma are likely to occur after operation, and excessive stretching of both sides after suture will lead to thinning of common bile duct diameter and stenosis. In addition, biliary reconstruction techniques such as bile duct transposition techniques and bridging anastomosis mediated by the gallbladder (31, 32), have also failed to be widely used because of more postoperative biliary complications. End-to-end biliary anastomosis is preferred increasingly this year due to its ability to maintain the normal anatomical relationship of the biliary system, facilitate endoscopy or treatment of biliary complications, and provide a shorter operation time. In this study, we utilized end-to-end biliary anastomosis.

There is a close relationship between the diameter of biliary anastomosis and the occurrence of biliary complications (3). Zhou et al. (33) analyzed the risk factors of biliary complications in 258 orthotopic liver transplant recipients by univariate analysis and logistic multivariate regression model, and the results showed that biliary tract and biliary anastomotic diameter were closely related to the occurrence of biliary complications after liver transplantation. Studies have shown that a donor to recipient biliary diameter ratio greater than 3:1 significantly increases the incidence of biliary stricture, and choledochojejunostomy is recommended for a ratio greater than 3:1 (34). The diameter of the donor liver, recipient biliary tract, and their degree of compatibility are critical factors in determining the anastomotic method and a significant predictor of biliary anastomotic stricture (35, 36).

Biliary reconstruction was performed in our center using the cystic duct plasty technique. On the one hand, the cystic duct is not foreign body, and it is close and good to the common bile duct, without postoperative necrosis and stenosis due to ischemia, on the other hand, the cystic duct itself is connected to the common bile duct, which can increase the diameter of the common bile duct and prevent anastomotic stenosis. Furthermore, performing end-to-end anastomosis following cystic duct plasty maintains the native anatomical relationship and reduces the risk of complications associated with choledochojejunostomy, which is more in line with biliary hydrodynamics. The results of our study also showed that for patients with thin common bile duct diameter or large difference in the proportion of donor and recipient common bile ducts, the use of cystic duct plasty technique to reconstruct the biliary tract can effectively reduce the overall incidence of postoperative biliary complications and anastomotic stricture.

There are shortcomings in this study. First, this study is a retrospective study, and the conclusion remains to be confirmed by further multicenter randomized controlled studies. Second, patients in the control group of this study had direct end-to-end anastomosis without T-tube placement or other techniques to prevent biliary complications, so it is not clear whether cystic duct plasty is superior to other techniques in this study. Third, we could not further analyze the results of various plasty procedures based on donor factors, because the personal information of donors is more private and there are difficulties in obtaining them.

To sum up, employing the cystic duct plasty technique to reconstruct the common bile duct appears to be a secure option for liver transplantation in patients with narrow common bile duct diameters or significant mismatch in the donor-recipient common bile duct proportions.

## Data Availability

The raw data supporting the conclusions of this article will be made available by the authors, without undue reservation.

## References

[1] Sapisochin G Bruix J. Liver transplantation for hepatocellular carcinoma: outcomes and novel surgical approaches. Nat Rev Gastroenterol Hepatol. (2017) 14(4):203–17. 10.1038/nrgastro.2016.193 28053342

[2] Hampe T Dogan A Encke J Mehrabi A Schemmer P Schmidt J Biliary complications after liver transplantation. Clin Transplant. (2006) 20(Suppl 17):93–6. 10.1111/j.1399-0012.2006.00607.x 17100708

[3] Kochhar G Parungao JM Hanouneh IA Parsi MA. Biliary complications following liver transplantation. World J Gastroenterol. (2013) 19(19):2841–6. 10.3748/wjg.v19.i19.2841 23704818 PMC3660810

[4] Moy BT Birk JW. A review on the management of biliary complications after orthotopic liver transplantation. J Clin Transl Hepatol. (2019) 7(1):61–71. 10.14218/JCTH.2018.00028 30944822 PMC6441650

[5] Roos FJM Poley JW Polak WG Metselaar HJ. Biliary complications after liver transplantation; recent developments in etiology, diagnosis and endoscopic treatment. Best Pract Res Clin Gastroenterol. (2017) 31(2):227–35. 10.1016/j.bpg.2017.04.002 28624111

[6] Senter-Zapata M Khan AS Subramanian T Vachharajani N Dageforde LA Wellen JR Patient and graft survival: biliary complications after liver transplantation. J Am Coll Surg. (2018) 226(4):484–94. 10.1016/j.jamcollsurg.2017.12.039 29360615

[7] Chang JH Lee I Choi MG Han SW. Current diagnosis and treatment of benign biliary strictures after living donor liver transplantation. World J Gastroenterol. (2016) 22(4):1593–606. 10.3748/wjg.v22.i4.1593 26819525 PMC4721991

[8] Wan P Yu X Xia Q. Operative outcomes of adult living donor liver transplantation and deceased donor liver transplantation: a systematic review and meta-analysis. Liver Transpl. (2014) 20(4):425–36. 10.1002/lt.23836 24478109

[9] 中华医学会器官移植学分会. 尸体器官捐献供体及器官评估和维护规范 (2019版) 器官移植. Organ Transplant. (2019) 10(3):253–62. 10.3969/j.issn.1674-7445.2019.03.006

[10] Zheng SS Xu X Wu J Chen J Wang WL Zhang M Liver transplantation for hepatocellular carcinoma: hangzhou experiences. Transplantation. (2008) 85(12):1726–32. 10.1097/TP.0b013e31816b67e4 18580463

[11] Nemes B Gaman G Doros A. Biliary complications after liver transplantation. Expert Rev Gastroenterol Hepatol. (2015) 9(4):447–66. 10.1586/17474124.2015.967761 25331256

[12] Mateo R Cho Y Singh G Stapfer M Donovan J Kahn J Risk factors for graft survival after liver transplantation from donation after cardiac death donors: an analysis of optn/unos data. Am J Transplant. (2006) 6(4):791–6. 10.1111/j.1600-6143.2006.01243.x 16539637

[13] Foley DP Fernandez LA Leverson G Chin LT Krieger N Cooper JT Donation after cardiac death: the university of Wisconsin experience with liver transplantation. Ann Surg. (2005) 242(5):724–31. 10.1097/01.sla.0000186178.07110.92 16244547 PMC1409855

[14] Skaro AI Jay CL Baker TB Wang E Pasricha S Lyuksemburg V The impact of ischemic cholangiopathy in liver transplantation using donors after cardiac death: the untold story. Surgery. (2009) 146(4):543–52; discussion 52–3. 10.1016/j.surg.2009.06.052 19789011 PMC2790600

[15] de Vera ME Lopez-Solis R Dvorchik I Campos S Morris W Demetris AJ Liver transplantation using donation after cardiac death donors: long-term follow-up from a single center. Am J Transplant. (2009) 9(4):773–81. 10.1111/j.1600-6143.2009.02560.x 19344466

[16] Jay C Ladner D Wang E Lyuksemburg V Kang R Chang Y A comprehensive risk assessment of mortality following donation after cardiac death liver transplant—an analysis of the national registry. J Hepatol. (2011) 55(4):808–13. 10.1016/j.jhep.2011.01.040 21338639 PMC3177011

[17] Mourad MM Algarni A Liossis C Bramhall SR. Aetiology and risk factors of ischaemic cholangiopathy after liver transplantation. World J Gastroenterol. (2014) 20(20):6159–69. 10.3748/wjg.v20.i20.6159 24876737 PMC4033454

[18] Cannistra M Ruggiero M Zullo A Gallelli G Serafini S Maria M Hepatic ischemia reperfusion injury: a systematic review of literature and the role of current drugs and biomarkers. Int J Surg. (2016) 33(Suppl 1):S57–70. 10.1016/j.ijsu.2016.05.050 27255130

[19] Ohtani O Kikuta A Ohtsuka A Taguchi T Murakami T. Microvasculature as studied by the microvascular corrosion casting/scanning electron microscope method. I. Endocrine and digestive system. Arch Histol Jpn. (1983) 46(1):1–42. 10.1679/aohc.46.1 6347120

[20] Yamamoto K Sherman I Phillips MJ Fisher MM. Three-dimensional observations of the hepatic arterial terminations in rat, hamster and human liver by scanning electron microscopy of microvascular casts. Hepatology. (1985) 5(3):452–6. 10.1002/hep.1840050318 3997074

[21] Croome KP Lee DD Keaveny AP Taner CB. Improving national results in liver transplantation using grafts from donation after cardiac death donors. Transplantation. (2016) 100(12):2640–7. 10.1097/TP.0000000000001483 27861295

[22] Croome KP. Donation after circulatory death: potential mechanisms of injury and preventative strategies. Semin Liver Dis. (2020) 40(3):256–63. 10.1055/s-0040-1709487 32557479

[23] Kaldas FM Korayem IM Russell TA Agopian VG Aziz A DiNorcia J Assessment of anastomotic biliary complications in adult patients undergoing high-acuity liver transplant. JAMA Surg. (2019) 154(5):431–9. 10.1001/jamasurg.2018.5527 30758485 PMC6537782

[24] Craig EV Heller MT. Complications of liver transplant. Abdom Radiol. (2021) 46(1):43–67. 10.1007/s00261-019-02340-5 31797026

[25] Kadaba RS Bowers KA Khorsandi S Hutchins RR Abraham AT Sarker SJ Complications of biliary-enteric anastomoses. Ann R Coll Surg Engl. (2017) 99(3):210–5. 10.1308/rcsann.2016.0293 27659373 PMC5450270

[26] Bettschart V Clayton RA Parks RW Garden OJ Bellamy CO. Cholangiocarcinoma arising after biliary-enteric drainage procedures for benign disease. Gut. (2002) 51(1):128–9. 10.1136/gut.51.1.128 12077105 PMC1773262

[27] Weiss S Schmidt SC Ulrich F Pascher A Schumacher G Stockmann M Biliary reconstruction using a side-to-side choledochocholedochostomy with or without T-tube in deceased donor liver transplantation: a prospective randomized trial. Ann Surg. (2009) 250(5):766–71. 10.1097/SLA.0b013e3181bd920a 19809299

[28] Scatton O Meunier B Cherqui D Boillot O Sauvanet A Boudjema K Randomized trial of choledochocholedochostomy with or without a T tube in orthotopic liver transplantation. Ann Surg. (2001) 233(3):432–7. 10.1097/00000658-200103000-00019 11224633 PMC1421262

[29] Sun N Zhang J Li X Zhang C Zhou X Zhang C. Biliary tract reconstruction with or without T-tube in orthotopic liver transplantation: a systematic review and meta-analysis. Expert Rev Gastroenterol Hepatol. (2015) 9(4):529–38. 10.1586/17474124.2015.1002084 25583036

[30] Neuhaus P Blumhardt G Bechstein WO Steffen R Platz KP Keck H. Technique and results of biliary reconstruction using side-to-side choledochocholedochostomy in 300 orthotopic liver transplants. Ann Surg. (1994) 219(4):426–34. 10.1097/00000658-199404000-00014 8161269 PMC1243160

[31] Evans RA Raby ND O'Grady JG Karani JB Nunnerley HB Calne RY Biliary complications following orthotopic liver transplantation. Clin Radiol. (1990) 41(3):190–4. 10.1016/s0009-9260(05)80966-3 2323165

[32] Carmody IC Romano J Bohorquez H Bugeaud E Bruce DS Cohen AJ Novel biliary reconstruction techniques during liver transplantation. Ochsner J. (2017) 17(1):42–5. 28331447 PMC5349635

[33] Luo Z Chen L Chen J Li X Zhou J. Diagnosis and treatment of biliary complications after liver transplantation: analysis of 258 cases. Nan Fang Yi Ke Da Xue Xue Bao. (2014) 34(5):709–12. 24849442

[34] Leal-Leyte P McKenna GJ Ruiz RM Anthony TL Saracino G Testa G Eversion bile duct anastomosis: a safe alternative for bile duct size discrepancy in deceased donor liver transplantation. Liver Transpl. (2018) 24(8):1011–8. 10.1002/lt.25075 29637692

[35] Davidson BR Rai R Kurzawinski TR Selves L Farouk M Dooley JS Prospective randomized trial of end-to-end versus side-to-side biliary reconstruction after orthotopic liver transplantation. Br J Surg. (1999) 86(4):447–52. 10.1046/j.1365-2168.1999.01073.x 10215812

[36] Paes-Barbosa FC Massarollo PC Bernardo WM Ferreira FG Barbosa FK Raslan M Systematic review and meta-analysis of biliary reconstruction techniques in orthotopic deceased donor liver transplantation. J Hepatobiliary Pancreat Sci. (2011) 18(4):525–36. 10.1007/s00534-010-0346-5 21127915

