# Corrigendum: Application of cholecystic duct plasty in the prevention of biliary complications following orthotopic liver transplantation

**DOI:** 10.3389/fsurg.2023.1297070

**Published:** 2023-10-09

**Authors:** Jing Wang, Song-ping Cui, Shao-cheng Lyu, Qing Chen, Jin-can Huang, Han-xuan Wang, Qiang He, Ren Lang

**Affiliations:** ^1^ Department of Hepatobiliary and Pancreaticosplenic Surgery, Beijing ChaoYang Hospital, Capital Medical University, Beijing, China; ^2^ Department of Thoracic Surgery, Beijing ChaoYang Hospital, Capital Medical University, Beijing, China

**Keywords:** orthotopic liver transplantation, biliary reconstruction, cholecystic duct plasty, biliary complication, prognosis

A Corrigendum on Application of cholecystic duct plasty in the prevention of biliary complications following orthotopic liver transplantation By Cui S-p, Lyu S-c, Wang J, Chen Q, Huang J-c, Wang H-x, He Q and Lang R (2023) Front. Surg. 10:1087327. doi: 10.3389/fsurg.2023.1087327

Error in Author List

In the published article, there was an error in the author list, and author [Jing Wang] was erroneously [ranked]. The corrected author list appears below.

Jing Wang^1,2†^, Song-ping Cui^1†^, Shao-cheng Lyu^1†^, Qing Chen^1^, Jin-can Huang^1^, Han-xuan Wang^1^, Qiang He^1^ and Ren Lang^1^*

The authors apologize for this error and state that this does not change the scientific conclusions of the article in any way. The original article has been updated.

